# Varietal Susceptibility of Yellow Onions to Blanching and Its Impact on Probiotic Fermentation

**DOI:** 10.3390/molecules30143002

**Published:** 2025-07-17

**Authors:** Katarzyna Grzelak-Błaszczyk, Robert Klewicki, Sylwia Ścieszka, Lidia Piekarska-Radzik, Michał Sójka, Michalina Jaszczak, Elżbieta Klewicka, Bartosz Fotschki, Jerzy Juśkiewicz

**Affiliations:** 1 Institute of Food Technology and Analysis, Lodz University of Technology, Stefanowskiego 2/22, 90-537 Łódź, Poland; robert.klewicki@p.lodz.pl (R.K.); michal.sojka@p.lodz.pl (M.S.);; 2 Institute of Fermentation Technology and Microbiology, Lodz University of Technology, Wólczańska 171/173, 90-530 Łódź, Poland; sylwia.scieszka@p.lodz.pl (S.Ś.); lidia.piekarska-radzik@p.lodz.pl (L.P.-R.); elzbieta.klewicka@p.lodz.pl (E.K.); 3 Institute of Animal Reproduction and Food Research, Polish Academy of Sciences, ul. Trylińskiego 18, 10-683 Olsztyn, Poland; b.fotschki@pan.olsztyn.pl (B.F.); j.juskiewicz@pan.olsztyn.pl (J.J.)

**Keywords:** *Allium cepa*, variety, blanching, probiotic fermentation

## Abstract

The purpose of this study was to determine the impact of blanching various onion *(Allium cepa* L.) varieties on the process of lactic fermentation by probiotic strain *Levilactobacillus brevis* ŁOCK 0944. The materials for the research were twelve varieties of yellow onion: Venecia, Moondance, Sedona, Alonso, Hysky, Centro, Dormo, Hypark, Hybelle, Armstrong, EXP 2236, and Hysinger. We also studied the resulting changes in bioactive compound content. Acidic bacterial metabolites, the lactic acid bacteria count, and the polyphenol and carbohydrate contents were assessed in both raw onions and onions blanched at 60 °C, before and after fermentation. Onion varieties that showed morphological changes after blanching (Hysky, Centro, Dormo) demonstrated better growth of *L. brevis* and higher lactic acid production. Blanching loosened the tissue structure, reducing the carbohydrate content in the blanched and fermented onions, particularly Alonso, Centro, Dormo, and Hypark varieties. Although the combined process reduced the polyphenol content, four varieties showed no statistically significant differences, indicating variety-specific responses. The varying susceptibility of onion varieties to thermal treatment highlights the importance of selecting the appropriate variety for further processing.

## 1. Introduction

Lactic acid fermentation has been used for food preservation since ancient times. The process was initially developed to preserve food, rather than with the intention of creating new food products [1]. In Europe, interest in producing fermented products dates back to the early twentieth century. Fermentation using lactic acid bacteria offers an excellent way to enhance both the sensory qualities and nutritional value of plant-based foods, as well as meat and dairy products [2]. Advances in food technology and evolving consumer expectations are driving food producers to create new, organoleptically and nutritionally interesting products. Vegetables commonly preserved through pickling include olives, cucumbers, white and red cabbage, garlic, beets, green beans, green tomatoes, sweet and hot peppers, and even cauliflower and carrots. More than 20 different fermented vegetable products (vegetables and vegetable juices) are produced in Europe. The most popular and economically profitable products include pickled olives, cucumbers, and cabbage. Fermented products popular globally include Korean Kimchi, i.e., pickled Korean cabbage with the addition of horseradish and other vegetables, such as garlic, red pepper, green onion, and ginger [3,4].

Spontaneous lactic acid fermentation of plant materials is highly unpredictable, including in terms of the sensory characteristics of the final product. To ensure the production of food products with desirable sensory and distribution properties, controlled fermentation can be conducted using carefully selected microorganisms. In industrial fermentation practice, vegetables and juices preserved thermally by pasteurization and inoculation are used with selected bacterial cultures at concentrations of 5×10^6^ to 1×10^7^ CFU/mL [3].

Common onions (*Allium cepa* L.) are one of the oldest and most valuable plants. Onions are consumed all over the world, and global demand for onions is growing. Between 2008 and 2018, onion production increased by approximately 30% [5]. Onion is a nutritious and bioactive vegetable. Among the basic nutrients, carbohydrates (from 2.3 to 7 g/100 g f. w.) dominate, followed by protein (1–1.4 g/100 g f. w.) and fat (0.1–0.4 g/100 g f. w.) [6,7,8]. Onions also provide many valuable minerals and vitamins. Onions are famous primarily for their high vitamin C content (5.3–10 mg/100 g f. w.). Among the micronutrients, it mainly provides zinc, iron, manganese, and copper. Taking into account the macronutrients, onions are the most abundant in potassium, containing 80–140 mg/100 g f. w., and in sulfur, 50–65 mg/100 g f. w. [9]. This vegetable is a valuable source of many health-promoting substances, i.e., polyphenols and fructooligosaccharides (FOS). FOS are usually composed of 2, 3, and 4 fructosyl residues connected by β (2 → 1) bonds and one glucose residue. These are kestose (DP-3), nystose (DP-4), and fructofuranosylnystose (DP-5) [10]. These compounds are known for their ability to intensify the growth of lactic bacteria, which has a beneficial effect on the human body [11]. Epidemiological studies suggest that fructooligosaccharides improve the absorption of minerals and vitamins, reduce the frequency of constipation, improve stool consistency, make weight control more effective, and play an important role in the treatment of atopic dermatitis [12,13]. An important group of health-promoting compounds in onions are polyphenols. Their ranges from 297 to 1289 mg/100 g d.m. [6,14,15,16]. The dominant group of polyphenols in onions are flavonols, mainly quercetin and its glycosides. Quercetin compounds have antiallergic, antiviral, antiinflammatory, antiparasitic, and antibacterial properties and have protective effects against cardiovascular disease, cancer, and other chronic diseases [12,17,18]. Onions are used as ingredients in a variety of foods, including soups, sauces, salad dressings, and sausages [19].

Thermal treatment, i.e., blanching in hot water, is commonly used before many vegetable processing methods, such as drying, canning, or freezing. The main goals of blanching are the inactivation of enzymes (to prevent possible deterioration reactions), the reduction of microbial contamination (to extend shelf life), the elimination of air from tissues (to increase oxidative stability, protect substances susceptible to oxidation, and stabilize color), and the modification of cell structure (to soften the texture and even increase the drying speed) [19]. Thermal treatment of raw materials, especially plant materials, is one of the simplest ways to eliminate endogenous microbiota, such as *Enterobacteriaceae*, yeasts, and filamentous fungi, which are often pathogenic [20]. The thermal process eliminates vegetative cells of contaminating microorganisms and promotes the rapid domination of the environment by lactic acid bacteria during controlled fermentation. However, compounds, such as polyphenols (especially flavonoids), may be degraded, resulting in a decrease in antioxidant activity. Nonetheless, mild heat treatment of onions at 50–60 °C can preserve a large proportion of their polyphenols, without reducing their antioxidant activity [21].

There is growing recognition of the importance of food ingredients, including antioxidants, for human health. It is important to use appropriate processing methods, including blanching, to obtain products with significant contents of bioactive compounds. However, there is no information in the literature on the impact of combined blanching and fermentation processes on the contents of carbohydrates and phenolic compounds in fermented products. There is also a lack of research on the compositional characteristics of different onion varieties subjected to pre-blanching.

Here, we investigated the impact of medium-temperature heating pretreatment of *Allium cepa* L. onions submitted to lactic fermentation. The lactic fermentation process was carried out using the probiotic strain *Levilactobacillus brevis* ŁOCK 0944. We evaluated the concentration of primary bacterial metabolites, the number of bacteria, and the concentrations of polyphenols and carbohydrates in raw, fermented onions and onions blanched at 60 °C.

## 2. Results and Discussion

This study assessed the impact of onion blanching on the process of lactic fermentation performed using *L. brevis* ŁOCK 0944. For all onion varieties, except Dormo and Hypark, the samples showed the same appearance under macroscopic evaluation before and after fermentation. In the case of non-blanched Dormo and Hypark varieties, the fermentation liquid and onions turned pink after fermentation. However, in the case of Dormo and Hypark varieties in the samples fermented after blanching, no pink color was noted. Microscopic analysis and identification were performed using classical methods. The results revealed that the Dormo and Hypark varieties after fermentation were contaminated with *Rhodotorula* spp. yeasts, which are saprophytes and occur in the soil as endophytes of the plant rhizosphere [22]. While there has been extensive research on non-pathogenic *Rhodotorula* spp., studies on pathogenic strains are limited. Strains of *Rhodotorula mucilaginosa* have been characterized as opportunistic pathogens causing dermatitis, particularly in immunocompromised individuals [23]. This yeast was not present in the samples after blanching, which shows that blanching effectively limits the growth of the onion’s own microbiota (in this case, the contaminating microbiota, which was a yeast of the genus *Rhodotorula*).

For all onion varieties, the initial number of bacteria ranged from 6.0 to 6.6 log CFU/mL of liquid before fermentation (Table 1). After 96 h of fermentation, an increase in bacterial biomass of approximately 2 log CFU/mL was observed. In the case of four onion varieties (Hysky, Centro, Dormo, Hybelle), the number of lactic acid bacteria was statistically significantly higher in the blanched samples. For the remaining onion varieties, no statistically significant differences were observed in the number of lactic acid bacteria in the blanched samples. Moreover, in all cases, after lactic fermentation, a product was obtained with a lactic acid bacteria count above 10^8^ CFU/mL.

Table 2 shows the profile of acids synthesized by *L. brevis* ŁOCK 0944 during fermentation of raw and blanched onions. The total concentration of lactic acid in the liquid after fermentation of raw onions ranged from 3.6 g/L to 5.4 g/L. For blanched onions, the lactic acid concentrations ranged from 2.8 g/L to 6.1 g/L. A statistically significant difference between the concentrations of lactic acid after fermentation of raw and blanched onions was noted in the cases of the Hysky, Centro, and Dormo varieties. In the same samples, a higher number of *L. brevis* was recorded after fermentation. The total concentration of acetic acid in the liquid after fermentation of raw onions ranged from 72.5 mg/L to 110.4 mg/L. In blanched onions, the total concentration of acetic acid in the liquid after fermentation ranged from 53.3 mg/L to 101.8 mg/L. Blanching before fermentation resulted in a statistically significant reduction in the concentration of acetic acid in the following varieties: Moondance, Alonso, Centro, Hypark, and Armstrong. For the remaining varieties, no statistically significant differences in the concentration of acetic acid were observed.

The most desirable fraction of lactic acid in food is the L-fraction. The stereospecificity of lactic acid depends upon the selected lactic acid bacteria strain. Lactic acid produced by lactic acid bacteria exists in the form of its conjugate base, either L-lactate or D-lactate, at pH 7.4. It can be either obtained as an optically active form or produced as a racemic mixture [24]. During microbial fermentation, d- or l-lactic acid is produced [25]. l-lactic acid is most commonly produced by the enzyme l-lactate dehydrogenase (LDH), using NAD+ (nicotinamide adenine dinucleotide) found in bacteria [26,27]. The organisms that predominantly produce the l (+)-isomer are *L. delbrueckii, L. amylophilus, L. bavaricus, L. casei, L. maltaromicus*, and *L. salivarius*. Strains, such as *L. lactis*, *L. jensenii*, and *L. acidophilus*, produce either the d-isomer or mixtures of both [25]. In our research, *Levilactobacillus brevis* ŁOCK 0944 synthesized a racemic mixture of lactic acid during onion fermentation. The proportions of l- and d-lactic acid isomers varied, depending on the onion variety and pretreatment by blanching, following fermentation. For onion varieties Venecia, Hysky, and Hysinger, the share of L-lactic acid was higher (while d-lactic acid was lower) for samples blanched before fermentation (BF) than in the case of raw onion fermentation (F).

During thermal treatment, the cytoplasmic membranes and cell walls of the onion pulp cells may be damaged, leading to the release and extraction of cell contents. The consequence of this phenomenon was an increase in the availability of compounds, including carbohydrates and polyphenols, which can be used by lactic acid bacteria as a carbon source in the lactic fermentation process.

Our research analyzed the carbohydrate content of raw onions, fermented onions, and onions fermented after blanching (Table 3). Based on chromatographic analysis, it was estimated that the total carbohydrate content in fresh onions ranged from 4.4 to 8.03 g/100 g, depending on the variety. After fermentation, the carbohydrate content decreased by 10–23%, and after blanching and fermentation, it decreased by 14–20%. The analysis of the carbohydrate content confirms that blanching loosens the structure of the tissue, which results in lower carbohydrate content in the tissue of blanched and fermented onions, especially in the case of Alonso, Centro, Dormo, and Hypark varieties. This indicates that the blanching process releases carbohydrates into the environment, making them an excellent nutrient for lactic acid bacteria, which then have better conditions for growth. This is especially visible for the Centro and Dormo varieties. Moreover, the results show that some onion varieties seem more susceptible to heat treatment than others. This important finding allows for the proper selection of onion varieties based on their stability for further processing.

Table 4 shows the influence of blanching and fermentation on the polyphenol content in the various onion varieties. Pretreatment with blanching resulted in the fluctuating loss of these bioactive compounds. After fermentation without pretreatment, the polyphenol content ranged from 18.62 to 58.34 mg/100 g, depending on the onion variety. After the combined process, the polyphenol content ranged from 14.98 to 43.93 mg/100 g. The loss of polyphenols mainly affected quercetin diglycosides, which remained in only small amounts in the blanched and fermented onions. Quercetin monoglycosides were more resilient to blanching and fermentation. The amounts of quercetin-4′-glucoside in the fermented onions ranged from 11.35 to 34.62 mg/100 g, while in blanched and fermented onions, it ranged from 6.03 to 26.55 mg/100 g. Heat treatment led to the degradation of quercetin glycosides. The main product is quercetin aglycone. The issue of the effect of heat treatment of food on flavonols has been raised for a long time, based on the results of studies that have indicated that regular home processing (frying, boiling, baking) can have a significant effect on the content and composition of flavonols in plant tissues. Later studies confirmed that cooking onions can cause a significant decrease in flavonol glycosides, which is related to the structure of the glycoside [28] The stability of glycosides depends on the type and position of the sugar grouping [29].

The differences in the polyphenol content between the fermented onions without pretreatment and the blanched and fermented onions were statistically significant for most varieties of onions. However, the total polyphenol content largely depended on the variety, as some showed no statistically significant differences between blanched and untreated onions. The varieties that showed no statistically significant differences included Venecia, Moondance, Alonso, and Hysky. This clearly indicates that selecting the appropriate onion variety for industrial processing is extremely important in order to preserve as many of its health-promoting bioactive ingredients as possible.

**Figure 1:**
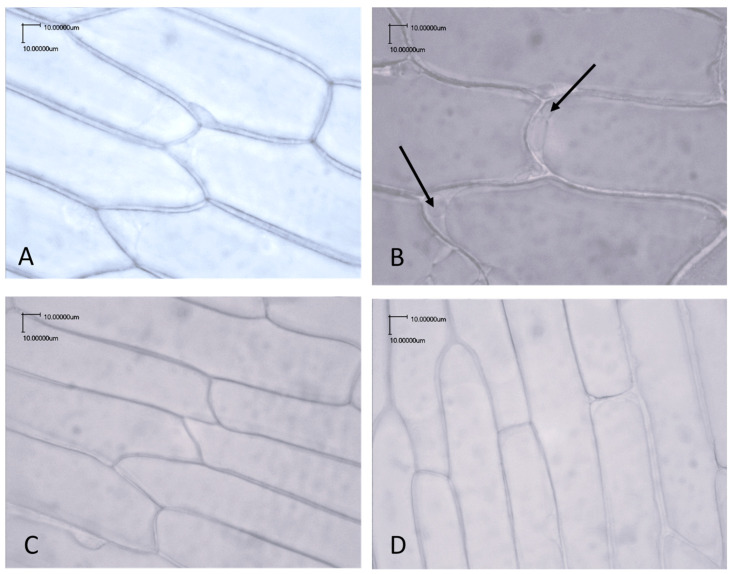
Light microscope images of raw (**A**,**C**) and blanched (**B**,**D**) onion skin cells. Photos (**A**,**B**), Dormo onion variety; photos (**C**,**D**), Armstrong onion variety. The arrow indicates changes caused by blanching, specifically detachment of the cytoplasmic membrane from the cell wall and the outflow of cell contents, visible as bubbles.

The pulp cells of the blanched and raw onions were compared by microscopic analysis using a light microscope. Given the fact that not all onion varieties showed increases in the number of lactic acid bacteria or increased acidification after blanching, we focused on the Hysky, Centro, and Dormo varieties, which exhibited more intense bacterial growth and a higher lactic acid concentration after blanching. The microscopic images showed changes in the pulp cells of the Hysky, Centro, and Dormo onion samples after blanching, including detachment of the cytoplasmic membrane from the cell wall and the outflow of cell contents, visible as bubbles (Figure 1B). In the unblanched pulp samples, no such changes were observed (Figure 1A). For comparison, microscopic images were also taken of onion varieties for which no statistically significant differences were observed, in terms of either lactic acid bacteria growth or souring. Again, no differences were observed in the morphology of the onion pulp cells before or after blanching (Figure 1C,D). Therefore, it can be concluded that onion bulbs have varying susceptibility to the blanching process.

## 3. Materials and Methods

### 3.1. Blanching

Twelve yellow onion varieties from a commercial plantation in central Poland (Bejo Poland) were used: Venecia, Moondance, Sedona, Alonso, Hysky, Centro, Dormo, Hypark, Hybelle, Armstrong, EXP 2236, and Hysinger. Three onions weighing 200–300 g were selected from each variety. The onions were cut into 16 parts, along the bulb (from the tail to the heel). For blanching, 50 ± 1 g of each of the onions were weighed, blanched at 60 °C for 3 min, strained, and then transferred to sterile jars. Both the raw and blanched onions were subjected to lactic acid fermentation. The contents of polyphenols and carbohydrates were determined in the raw, fermented, and blanched and fermented onions using HPLC.

### 3.2. Lactic Acid Fermentation

A container with 50 ± 1 g of onions and 70 mL of sterile physiological salt (0.9% *w*/*v*) was inoculated with 1% (*v*/*v*) inoculum prepared from an overnight culture of *Levilactobacillus brevis* ŁOCK 0944 (initiation density 10^6^ CFU/mL). Fermentation was carried out at 30 °C for 96 h. After this time, the onion was separated from the fermentation broth. The liquid was analyzed for the acid content and the number of lactic acid bacteria.

### 3.3. Microorganisms

A strain belonging to the genus *Levilactobacillus brevis*, marked with the symbols ŁOCK 0944, was used for the fermentation process. The strain is deposited with the Pure Culture Collection of Industrial Microorganisms (ŁOCK 105) in Łódź, Poland. *L. brevis* ŁOCK 0944 has a patent deposit number (B/00035) in the Polish Collection of Microorganisms. This bacterium was isolated from spontaneous plant silage (red beet) and has patented probiotic properties (number of patent: PL 235849 B1). The strain was stored in cryobanks at −20 °C. Before the experiment, the strain was activated by two passages in MRS broth (Merck, Darmstadt, Germany) at 30 °C for 24 h.

### 3.4. Determination of Polyphenols and Carbohydrates

The polyphenol content was determined according to the methodology described by Grzelak-Błaszczyk et al. (2023) [30]. The carbohydrate content was determined using high-performance liquid chromatography (HPLC). First, 2 g of the sample was weighed with an accuracy of 0.0001 g. Then, 0.2 g of calcium carbonate and 8 mL of distilled water were added. The solution was mixed, boiled, and kept at the boiling point for 3 min. The samples were cooled, quantitatively transferred to a 10 mL volumetric flask, topped up with water, and mixed. The extracts were centrifuged at 15,000 RCF for 10 min at 4 °C (MPW-360 R, Warsaw, Poland). The samples were desalted on a bed of cation exchanger and anion exchanger (1:2). The extract was analyzed on a liquid chromatograph (Knauer, Geretsried, Germany), with an RI detector and an Aminex HPX-87C calcium column. Separation was carried out in an isocratic system, with water as the mobile phase. The mobile phase flow rate was 0.5 mL/min. The volume of the dispensed sample was 20 μL. Identification of carbohydrates was based on standard substances: sucrose, glucose, fructose, and kestose (Merck, Darmstadt, Germany). The analysis was repeated twice for each sample.

### 3.5. Determination of Lactic and Acetic Acids

The concentrations of d- and l-lactic acid, as well as acetic acid, were determined using enzymatic tests by Megazyme (Bray, Ireland), following the manufacturer’s instructions. The fermentation liquid was filtered through a 0.22 μm filter (ALFATEC Technology, Przeźmierowo, Poland). The samples prepared in this way were stored at −20 °C prior to testing [30].

### 3.6. Determination of the Amount of Lactic Acid Bacteria in the Liquid After Onion Fermentation

The lactic acid bacteria count was determined according to the procedure described by Grzelak-Błaszczyk et al. (2023) [30]. After fermentation, the onions were vigorously shaken for 3 min to release the lactic acid bacteria adhering to the onion fragments. Then, 1 mL of the liquid was collected, and a series of tenfold dilutions were made. The samples were plated on Petri dishes with MRS agar (Merck, Darmstadt, Germany). The plates were incubated for 48 h at 30 °C. The results are presented as log colony-forming units (CFU) per mL.

### 3.7. Microscopic Analysis

Microscopic analysis of the raw and blanched onion peels was carried out using an Olympus CX41 light microscope (Olympus Corporation, Tokyo, Japan). An HDCE-X5 camera (Carton Optical Industries, Ltd., Tokyo, Japan) with ScopeImage 9.0 software was used for visualization. The total magnification of the images obtained was 400×.

### 3.8. Statistical Analysis

Statistical analysis was conducted using Statistica 10 (Stat Soft, Tulsa, OK, USA) software. The results were subjected to one-way Anova statistical analysis, *p* ≤ 0.05, and post hoc Duncan test.

## 4. Conclusions

This research investigated the influence of blanching on the morphology of onion skin cells. Different onion varieties were tested. Onion varieties in which morphological changes were observed after blanching at 60 °C, resulting in destruction of the outer cell membranes, were characterized by better growth of the probiotic strain *Levilactobacillus brevis* ŁOCK 0944 and higher lactic acid concentrations. This was related to improved access to nutrients for the bacteria. For the Hysky variety, the blanching process resulted in a higher number of lactic acid bacteria after fermentation, increased lactic acid content (more l-, less d- isomers), and no statistically significant differences in total polyphenol content. The results demonstrate that it is possible to determine the susceptibility of different onion varieties to the blanching process. This could enable identification of the most suitable onion varieties for technological processing, such as blanching and lactic fermentation.

## Figures and Tables

**Table 1:** Number of *Levilactobacillvs brevis* ŁOCK 0944 depending on the variety of onion and pretreatment by blanching.

Onion Variety	Pretreatment Conditions	Log (CFU/mL) ± SD Start	Log (CFU/mL) ± SD 96 h
Venecia	F	6.6 ± 0.16 ^a^	8.7 ± 0.04 ^b^*
	BF	6.6 ± 0.16 ^a^	8.6 ± 0.05 ^b^*
Moondance	F	6.6 ± 0.16 ^a^	8.9 ± 0.05 ^b^*
	BF	6.6 ± 0.16 ^a^	8.8 ± 0.34 ^b^*
Sedona	F	6.6 ± 0.16 ^a^	8.9 ± 0.17 ^b^*
	BF	6.6 ± 0.16 ^a^	8.5 ± 0.21 ^b^*
Alonso	F	6.6 ± 0.16 ^a^	8.9 ± 0.02 ^b^*
	BF	6.6 ± 0.16 ^a^	9.0 ± 0.09 ^b^*
Hysky	F	6.0 ± 0.12 ^a^	8.4 ± 0.00 ^b^*
	BF	6.0 ± 0.12 ^a^	8.9 ± 0.80 ^b#^
Centro	F	6.0 ± 0.12 ^a^	8.5 ± 0.13 ^b^*
	BF	6.0 ± 0.12 ^a^	9.0 ± 0.16 ^b#^
Dormo	F	6.0 ± 0.12 ^a^	8.3 ± 0.15 ^b^*
	BF	6.0 ± 0.12 ^a^	8.6 ± 0.05 ^b#^
Hypark	F	6.0 ± 0.12 ^a^	8.6 ± 0.06 ^b^*
	BF	6.0 ± 0.12 ^a^	8.8 ± 0.09 ^b^*
Hybelle	F	6.5 ± 0.02 ^a^	8.4 ± 0.03 ^b^*
	BF	6.5 ± 0.02 ^a^	8.7 ± 0.06 ^b#^
Armstrong	F	6.5 ± 0.02 ^a^	8.6 ± 0.00 ^b^*
	BF	6.5 ± 0.02 ^a^	8.9 ± 0.15 ^b^*
EXP 2236	F	6.5 ± 0.02 ^a^	8.6 ± 0.11 ^b^*
	BF	6.5 ± 0.02 ^a^	8.7 ± 0.00 ^b^*
Hysinger	F	6.5 ± 0.02 ^a^	8.6 ± 0.05 ^b^*
	BF	6.5 ± 0.02 ^a^	8.7 ± 0.02 ^b^*

^a,b^—statistical differences with respect to the initial sample (Start) in level; *^,#^ statistical differences between the fermented (F) and the fermented and blanched (BF) samples for the onion variety.

**Table 2:** Acidifying effect of *Levilactobacillvs brevis* ŁOCK 0944 bacteria depending on the variety of onion and pretreatment by blanching.

Onion Variety	Pretreatment Conditions	*Levilactobacillus brevis* ŁOCK 0944			
		Lactic Acid [g/L] ± SD	Fraction of D-Lactic Acid [%]	Fraction of L-Lactic Acid [%]	Acetic Acid [mg/L] ± SD
Venecia	F	4.8 ± 0.21 ^a^	53 ^A^	47 ^C^	73.6 ± 4.16 ^a^
	BF	3.9 ± 0.42 ^a^	37 ^B^	63 ^D^	81.4 ± 19.21 ^a^
Moondance	F	4.8 ± 0.53 ^a^	46 ^A^	54 ^C^	82.0 ± 0.36 ^a^
	BF	3.7 ± 0.28 ^a^	54 ^B^	46 ^D^	69.6 ± 3.28 ^b^
Sedona	F	4.5 ± 0.72 ^a^	56 ^A^	44 ^C^	88.5 ± 0.00 ^a^
	BF	2.8 ± 1.22 ^a^	54 ^A^	46 ^C^	71.6 ± 14.6 ^a^
Alonso	F	3.6 ± 0.26 ^a^	50 ^A^	50 ^C^	100.4 ± 9.00 ^a^
	BF	3.9 ± 0.29 ^a^	59 ^B^	41 ^D^	79.7 ± 16.2 ^b^
Hysky	F	4.9 ± 0.66 ^a^	48 ^A^	52 ^C^	98.5 ± 8.00 ^a^
	BF	6.1 ± 0.22 ^b^	28 ^B^	72 ^D^	84.1 ± 10.00 ^a^
Centro	F	4.9 ± 0.03 ^a^	63 ^A^	37 ^C^	72.5 ± 7.60 ^a^
	BF	5.2 ± 0.30 ^b^	61 ^A^	39 ^C^	57.3 ± 0.00 ^b^
Dormo	F	4.3 ± 0.58 ^a^	58 ^A^	42 ^C^	110.4 ± 4.51 ^a^
	BF	5.1 ± 0.18 ^b^	65 ^B^	35 ^D^	101.8 ± 8.40 ^a^
Hypark	F	4.7 ± 0.89 ^a^	51 ^A^	49 ^C^	100.0 ± 10.90 ^a^
	BF	3.7 ± 0.57 ^a^	56 ^A^	44 ^C^	70.5 ± 8.51 ^b^
Hybelle	F	5.0 ± 0.00 ^a^	55 ^A^	45 ^C^	79.6 ± 0.65 ^a^
	BF	4.7 ± 0.70 ^a^	53 ^A^	47 ^C^	72.3 ± 7.37 ^a^
Armstrong	F	5.4 ± 0.02 ^a^	54 ^A^	46 ^C^	84.2 ± 9.42 ^a^
	BF	5.6 ± 0.45 ^a^	54 ^A^	46 ^C^	63.1 ± 3.45 ^b^
EXP 2236	F	4.9 ± 0.01 ^a^	51 ^A^	49 ^C^	75.2 ± 0.60 ^a^
	BF	5.1 ± 0.34 ^a^	56 ^A^	44 ^C^	82.2 ± 8.95 ^a^
Hysinger	F	5.0 ± 0.25 ^a^	60 ^A^	40 ^C^	89.9 ± 4.5 ^a^
	BF	6.0 ± 0.47 ^a^	53 ^B^	47 ^D^	77.6 ± 10.43 ^a^

^a,b^—statistical differences within the variety between fermented onions (F) and blanched and fermented onions (BF); ^A,B^—statistical differences within the variety between fermented onion (F) and blanched and fermented onion (BF) for the concentration of D-lactic acid; ^C,D^—statistical differences within the variety between fermented onion (F) and blanched and fermented onion (BF) for the concentration of L-lactic acid.

**Table 3:** Carbohydrate content depending on the variety of onions and pretreatment with blanching.

Onion Variety	Pretreatment Conditions	Σ Carbohydrates	Σ FOS	Saccharose	Glucose	Fructose
		g/100 g f. w.				
Venecia	R	4.40 ± 0.47 ^b^	0.15 ± 0.01 ^a^	0.34 ± 0.05 ^b^	1.98 ± 0.21 ^b^	1.93 ± 0.20 ^b^
	F	0.69 ± 0.06 ^a^	0.18 ± 0.01 ^a^	0.06 ± 0.01 ^a^	0.20 ± 0.04 ^a^	0.24 ± 0.01 ^a^
	BF	0.67 ± 0.08 ^a^	0.20 ± 0.03 ^a^	0.06 ± 0.01 ^a^	0.19 ± 0.04 ^a^	0.23 ± 0.05 ^a^
Moondance	R	5.74 ± 0.34 ^b^	0.38 ± 0.02 ^a^	0.48 ± 0.03 ^b^	2.55 ± 0.15 ^c^	2.42 ± 0.14 ^b^
	F	0.82 ± 0.02 ^a^	0.35 ± 0.00 ^a^	0.08 ± 0.00 ^a^	0.18 ± 0.03 ^a^	0.20 ± 0.01 ^a^
	BF	0.90 ± 0.09 ^a^	0.31 ± 0.02 ^b^	0.09 ± 0.02 ^a^	0.31 ± 0.04 ^b^	0.19 ± 0.01 ^a^
Sedona	R	6.20 ± 0.09 ^c^	0.58 ± 0.01 ^b^	0.81 ± 0.00 ^b^	2.65 ± 0.05 ^b^	2.16 ± 0.03 ^b^
	F	0.65 ± 0.14 ^a^	0.24 ± 0.02 ^a^	0.06 ± 0.02 ^a^	0.15 ± 0.05 ^a^	0.20 ± 0.09 ^a^
	BF	0.86 ± 0.05 ^b^	0.26 ± 0.00 ^a^	0.07 ± 0.01 ^a^	0.25 ± 0.06 ^a^	0.27 ± 0.02 ^a^
Alonso	R	8.03 ± 0.15 ^c^	1.86 ± 0.05 ^c^	1.61 ± 0.03 ^b^	2.45 ± 0.07 ^b^	2.11 ± 0.00 ^b^
	F	1.22 ± 0.08 ^a^	0.53 ± 0.01 ^a^	0.17 ± 0.05 ^a^	0.29 ± 0.01 ^a^	0.23 ± 0.01 ^a^
	BF	1.09 ± 0.01 ^b^	0.41 ± 0.00 ^b^	0.19 ±0.01 ^a^	0.31 ± 0.06 ^a^	0.18 ± 0.05 ^a^
Hysky	R	7.40 ± 0.28 ^b^	1.87 ± 0.12 ^b^	1.40 ± 0.03 ^c^	2.31 ± 0.14 ^c^	1.83 ± 0.22 ^b^
	F	1.08 ± 0.32 ^a^	0.41 ± 0.14 ^a^	0.15 ± 0.05 ^a^	0.26 ± 0.02 ^a^	0.25 ± 0.10 ^a^
	BF	1.30 ± 0.21 ^a^	0.50 ± 0.06 ^a^	0.27 ± 0.04 ^b^	0.33 ± 0.06 ^b^	0.19 ± 0.05 ^a^
Centro	R	6.71 ± 0.04 ^c^	1.40 ± 0.15 ^c^	1.36 ± 0.01 ^b^	2.17 ± 0.02 ^c^	1.78 ± 0.09 ^c^
	F	1.28 ± 0.00 ^a^	0.55 ± 0.00 ^a^	0.16 ± 0.04 ^a^	0.25 ± 0.01 ^a^	0.32 ± 0.06 ^a^
	BF	1.05 ± 0.04 ^b^	0.47 ± 0.03 ^b^	0.21 ±0.01 ^a^	0.29 ± 0.01 ^b^	0.08 ± 0.01 ^b^
Dormo	R	7.18 ± 0.46 ^c^	2.43 ± 0.15 ^c^	1.40 ± 0.05 ^c^	1.67 ± 0.12 ^b^	1.67 ± 0.14 ^c^
	F	1.53 ± 0.04 ^a^	0.60 ± 0.08 ^a^	0.38 ± 0.03 ^a^	0.27 ± 0.00 ^a^	0.28 ± 0.06 ^a^
	BF	1.02 ± 0.11 ^b^	0.41 ± 0.00 ^b^	0.23 ± 0.04 ^b^	0.27 ± 0.03 ^a^	0.11 ± 0.03 ^b^
Hypark	R	6.75 ± 0.25 ^c^	1.60 ± 0.12 ^c^	1.57 ± 0.02 ^c^	1.97 ± 0.06 ^b^	1.61 ± 0.05 ^c^
	F	1.48 ± 0.12 ^a^	0.53 ± 0.05 ^a^	0.24 ± 0.02 ^a^	0.37 ± 0,02^a^	0.34 ± 0.03 ^a^
	BF	1.18 ± 0.02 ^b^	0.45 ± 0.02 ^b^	0.19 ± 0.00 ^b^	0.38 ± 0.03 ^a^	0.15 ± 0.01 ^b^
Hybelle	R	6.47 ± 0.06 ^b^	0.93 ± 0.12 ^b^	1.46 ± 0.05 ^b^	2.14 ± 0.03 ^c^	1.94 ± 0.08 ^c^
	F	1.00 ± 0.21 ^a^	0.26 ± 0.13 ^a^	0.15 ± 0.05 ^a^	0.28 ± 0.01 ^a^	0.30 ± 0.02 ^a^
	BF	0.98 ± 0.05 ^a^	0.24 ± 0.00 ^a^	0.19 ± 0.01 ^a^	0.36 ± 0.04 ^b^	0.19 ± 0.00 ^b^
Armstrong	R	6.63 ± 0.19 ^b^	1.08 ± 0.02 ^b^	1.67 ± 0.03 ^b^	2.08 ± 0.07 ^b^	1.79 ± 0.11 ^b^
	F	1.10 ± 0.21 ^a^	0.34 ± 0.08 ^a^	0.23 ± 0.05 ^a^	0.30 ± 0.04 ^a^	0.24 ± 0.04 ^a^
	BF	1.10 ± 0.09 ^a^	0.35 ± 0.07 ^a^	0.22 ± 0.03 ^a^	0.32 ± 0.01 ^a^	0.21 ± 0.00 ^a^
EXP 2236	R	6.28 ± 0.40 ^b^	1.25 ±0.17 ^b^	1.46 ± 0.12 ^b^	2.10 ± 0.09 ^b^	1.48 ± 0.02 ^c^
	F	1.30 ± 0.16 ^a^	0.40 ± 0.08 ^a^	0.25 ± 0.03 ^a^	0.38 ± 0.02 ^a^	0.27 ± 0.03 ^a^
	BF	1.02 ± 0.30 ^a^	0.37 ± 0.09 ^a^	0.19 ± 0.11 ^a^	0.30 ± 0.07 ^a^	0.16 ± 0.04 ^b^
Hysinger	R	6.34 ± 0.50 ^b^	1.42 ± 0.01 ^c^	1.35 ± 0.09 ^b^	2.08 ± 0.21 ^b^	1.49 ± 0.20 ^c^
	F	1.45 ± 0.30 ^a^	0.59 ± 0.00 ^a^	0.32 ± 0.01 ^a^	0.29 ± 0.01 ^a^	0.25 ± 0.01 ^a^
	BF	1.26 ± 0.00 ^a^	0.49 ± 0.05 ^b^	0.29 ± 0.02 ^a^	0.31 ± 0.05 ^a^	0.17 ± 0.02 ^b^

^a,b,c^—statistical differences within the variety between raw onion (R), fermented (F), and blanched and fermented onion (BF).

**Table 4:** Polyphenol content depending on the variety of onion and pretreatment by blanching.

Onion Variety	Pretreatment Conditions	Q-4′G	QM	QD	Q	Σ P
		mg/100 g f. w.				
Venecia	R	19.55 ± 2.59 ^a^	22.21 ± 2.80 ^a^	17.72 ± 2.11 ^b^	0.00 ± 0.00 ^b^	40.09 ± 4.91 ^b^
	F	17.67 ± 2.05 ^a^	18.60 ± 2.36 ^a^	0.23 ± 0.03 ^a^	10.46 ± 0.50 ^a^	32.57 ± 3.89 ^ab^
	BF	12.09 ± 2.25 ^b^	12.83 ± 2.51 ^b^	0.15 ± 0.01 ^a^	12.55 ± 3.46 ^a^	27.48 ± 7.03 ^a^
Moondance	R	24.25 ± 1.13 ^a^	29.28 ± 1.30 ^a^	16.81 ± 0.56 ^b^	0.00 ± 0.00 ^b^	46.25 ± 1.85 ^b^
	F	12.39 ± 1.12 ^b^	13.44 ± 1.32 ^b^	0.14 ± 0.02 ^a^	5.40 ± 0.84 ^a^	20.17 ± 2.52 ^a^
	BF	9.11 ± 1.85 ^c^	9.72 ± 1.91 ^c^	0.14 ± 0.04 ^a^	7.66 ± 1.78 ^a^	18.61 ± 3.92 ^a^
Sedona	R	24.74 ± 1.60 ^a^	27.16 ± 1.70 ^a^	17.46 ± 1.40 ^b^	0.00 ± 0.00 ^a^	44.85 ± 3.30 ^a^
	F	11.35 ± 0.94 ^b^	12.10 ± 1.04 ^b^	0.09 ± 0.01 ^a^	5.53 ± 0.36 ^b^	18.62 ± 1.51 ^b^
	BF	6.03 ± 0.52 ^c^	6.22 ± 0.78 ^c^	0.06 ± 0.02 ^a^	7.78 ± 0.61 ^c^	14.98 ± 1.69 ^c^
Alonso	R	26.37 ± 2.50 ^a^	29.36 ± 2.70 ^ab^	27.34 ± 0.50 ^b^	0.00 ± 0.00 ^b^	56.94 ± 4.50 ^b^
	F	31.89 ± 0.59 ^b^	33.34 ± 0.73 ^a^	0.24 ± 0.34 ^a^	9.76 ± 1.51 ^a^	44.51 ± 1.32 ^a^
	BF	26.55 ± 2.14 ^a^	27.79 ± 2.19 ^b^	0.29 ± 0.02 ^a^	12.14 ± 2.77 ^a^	41.45 ± 2.08 ^a^
Hysky	R	29.22 ± 1.80 ^a^	32.11 ± 2.30 ^a^	25.22 ± 1.20 ^b^	0.00 ± 0.00 ^b^	57.54 ± 3.50 ^b^
	F	22.09 ± 2.85 ^b^	22.90 ± 2.92 ^b^	0.28 ± 0.06 ^a^	12.99 ± 0.34 ^a^	37.19 ± 3.35 ^a^
	BF	13.70 ± 3.36 ^c^	14.12 ± 3.48 ^c^	0.20 ± 0.00 ^a^	14.39 ±1.80 ^a^	29.70 ± 5.48 ^a^
Centro	R	46.69 ± 0.70 ^a^	49.21 ± 0.76 ^a^	45.43 ± 1.37 ^b^	0.00 ± 0.00 ^b^	95.46 ± 2.12 ^c^
	F	34.62 ± 7.44 ^b^	35.55 ± 7.62 ^b^	0.71 ± 0.07 ^a^	20.73 ± 0.67 ^a^	58.34 ± 8.35 ^a^
	BF	23.30 ± 0.81 ^c^	23.78 ± 0.88 ^c^	0.45 ± 0.05 ^a^	18.92 ± 4.04 ^a^	43.93 ± 5.07 ^b^
Dormo	R	45.11 ± 1.47 ^a^	51.34 ± 1.73 ^a^	33.88 ± 0.70 ^b^	0.00 ± 0.00 ^b^	85.62 ± 2.41 ^c^
	F	21.62 ± 3.93 ^b^	22.35 ± 4.10 ^b^	0.32 ± 0.06 ^a^	18.12 ± 1.93 ^a^	42.36 ± 2.34 ^a^
	BF	11.32 ± 0.86 ^c^	11.63 ± 0.88 ^c^	0.22 ± 0.02 ^a^	20.69 ± 5.59 ^a^	33.70 ± 5.07 ^b^
Hypark	R	35.43 ± 0.13 ^a^	39.06 ± 0.26 ^a^	25.91 ± 2.15 ^b^	0.00 ± 0.00 ^c^	65.22 ± 2.45 ^c^
	F	27.65 ± 2.02 ^b^	28.91 ± 2.10 ^b^	0.32 ± 0.02 ^a^	12.20 ± 2.36 ^a^	42.63 ± 4.49 ^a^
	BF	12.96 ± 2.20 ^c^	13.47 ±2.28 ^c^	0.20 ± 0.02 ^a^	18.38 ± 0.88 ^b^	33.55 ± 1.32 ^b^
Hybelle	R	27.24 ± 1.70 ^a^	32.70 ± 1.90 ^a^	27.15 ± 1.70 ^b^	0.00 ± 0.00 ^b^	60.21 ± 4.20 ^a^
	F	30.03 ± 1.71 ^a^	31.38 ± 1.82 ^a^	0.42 ± 0.05 ^a^	19.31 ± 1.79 ^a^	52.90 ± 3.93 ^a^
	BF	15.72 ± 1.24 ^b^	16.24 ± 1.27 ^b^	0.22 ± 0.03 ^a^	15.99 ± 3.58 ^a^	33.81 ± 5.97 ^b^
Armstrong	R	29.44 ± 2.30 ^a^	30.93 ± 2.70 ^a^	27.87 ± 2.50 ^b^	0.00 ± 0.00 ^b^	59.19 ± 4.90 ^c^
	F	26.10 ± 2.39 ^a^	26.71 ± 2.49 ^a^	0.48 ± 0.12 ^a^	11.89 ± 0.69 ^a^	39.78 ± 3.35 ^a^
	BF	17.92 ± 1.26 ^b^	18.35 ± 1.27 ^b^	0.38 ± 0.09 ^a^	13.01 ± 0.66 ^a^	32.33 ± 3.11 ^b^
EXP 2236	R	32.92 ± 1.80 ^a^	34.18 ± 1.74 ^a^	34.89 ± 2.27 ^b^	0.00 ± 0.00 ^b^	69.55 ± 4.01 ^c^
	F	28.89 ± 2.51 ^a^	29.39 ± 2.52 ^a^	0.57 ± 0.03 ^a^	25.46 ± 3.34 ^a^	56.37 ± 5.93 ^a^
	BF	13.92 ± 4.29 ^b^	14.12 ± 4.32 ^b^	0.31 ± 0.09 ^a^	23.72 ± 4.12 ^a^	38.80 ± 2.93 ^b^
Hysinger	R	29.60 ± 1.96 ^c^	32.22 ± 2.17 ^c^	24.24 ± 1.59 ^b^	0.00 ± 0.00 ^c^	56.75 ± 3.77 ^c^
	F	19.66 ± 2.38 ^a^	20.20 ± 2.45 ^a^	0.29 ± 0.04 ^a^	11.29 ± 0.10 ^a^	32.63 ± 2.59 ^a^
	BF	12.48 ± 1.98 ^b^	12.84 ± 2.05 ^b^	0.20 ± 0.03 ^a^	13.22 ± 0.60 ^b^	27.10 ± 2.72 ^b^

^a,b,c^—statistical differences within the variety among raw onion (R), fermented (F), and blanched and fermented onion (BF). Q-4′G-quercetin-4′-glucoside, QM-quercetin monoglyosides, QD-quercetin diglycosides, Q-quercetin, P-polyphenols.

## Data Availability

The research data supporting this study have been deposited in the Open Research Data Repository of Lodz University of Technology and are accessible at: https://doi.org/10.34658/RDB.5OJVAT.

## References

[1] Prado F.C., Parada J.L., Pandey A., Soccol C.R. (2008). Trends in non-dairy probiotic beverages. Food Res. Int..

[2] Daba G.M., Elkhateeb W.A. (2020). Bacteriocins of lactic acid bacteria as biotechnological tools in food and pharmaceuticals: Current applications and future prospects. Biocatal. Agric. Biotechnol..

[3] Karovicova J., Kohajdova Z. (2003). Lactic acid fermented vegetable juices. Hortic. Sci..

[4] Caplice E., Fitzerald G.F. (1999). Food fermentation: Role of microorganisms in food production and preservation. Int. J. Food Microbiol..

[5] Vojvodić A., Šeremet D., Mandura A., Martinić A., Komes D. (2020). Onion solid waste as a potential source of functional food ingredients. Eng. Power..

[6] Benítez V., Mollá E., Martín-Cabrejas M.A., Aguilera Y., López-Andréu F.J., Cools K., Terry L.A., Esteban R.M. (2011). Characterization of industrial onion wastes (*Allium cepa* L.): Dietary fibre and bioactive compounds. Plant Foods Hum Nutr..

[7] Petropoulos S.A., Fernandes A., Barros L., Ferreira I.C.F.R., Ntatsi G. (2015). Morphological, nutritional and chemical description of vatikiotiko, an onion local landrace from Greece. Food Chem..

[8] Liguori L., Califano R., Albanese D., Raimo F., Crescitelli A., Di Matteo M. (2017). Chemical composition and antioxidant properties of five white onion (*Allium cepa* L.) *Landraces*. J. Food Qual..

[9] Bhattacharjee S., Sultana A., Sazzad M.H., Islam M.A., Ahtashom M.M. (2013). Analysis of the proximate composition and energy values of two varieties of onion (*Allium cepa* L.) bulbs of different origin: A comparative study. Int. J. Nutr. Food Sci..

[10] Grzelak K., Milala J., Krol B., Adamicki F., Badelek E. (2009). Content of quercetin glycosides and fructooligosaccharides in onion stored in cold room. Eur. Food Res. Technol..

[11] Sridevi V., Sumathi V., Guru Prasad M., Satish Kumar M. (2014). Fructooligosaccharides-type prebiotic: A Review. J. Res. Pharm..

[12] Costa G., Vasconcelos Q., Abreu G., Albuquerque A., Vilarejo J., Aragão G. (2020). Changes in nutrient absorption in children and adolescents caused by fructans, especially fructooligosaccharides and inulin. Arch. Pediatrie.

[13] Mainzer C., Le Guillou M., Vyumvuhore R., Chadoutaud B., Bordes S., Closs B. (2019). Clinical efficacy of oligofructans from Ophiopogon japonicus in reducing atopic dermatitis flare-ups in caucasian patients. Acta Derm. Venereol..

[14] Issa M., Aljoubbeh M. (2013). Total polyphenols, flavonoid content, kaempferol concetration and antioxidant activity of two onion syrian (spring and white). Int. J. Chemtech Res..

[15] Slimestad R., Fossen T., Vågen I.M. (2007). Onions: A source of unique dietary flavonoids. J. Agric. Food Chem..

[16] Tsanova-Savova S. (2011). Biologically active composition and health impact of allium cepa. Acta Med. Bulg..

[17] Mendonça R.D., Carvalhod N.C., Martin-Moreno J.M., Pimenta A.M., Lopes A.C.S., Gea A., Martinez-Gonzalez M.A., Bes-Rastrollo M. (2019). Total polyphenol intake, polyphenol subtypes and incidence of cardiovascular disease. Nutr. Metab. Cardiovasc. Dis..

[18] Yi J., Li S., Wang C., Cao N., Qu H., Cheng C., Wang Z., Wang L., Zhou L. (2019). Potential applications of polyphenols on main ncRNAs regulations as novel therapeutic strategy for cancer. Biomed. Pharmacother..

[19] Ren F., Perussello C.A., Zhang Z., Gaffney M.T., Kerryb J.P., Tiwari B.K. (2018). Enhancement of phytochemical content and drying efficiency of onions (*Allium cepa* L.) through blanching. J. Sci. Food Agric..

[20] Fan M., Rakotondrabe T.F., Chen G., Guo M. (2023). Advances in microbial analysis: Based on volatile organic compounds of microorganisms in food. Food Chem..

[21] Siddiq M., Roidoung S., Sogi D.S., Dolan K.D. (2013). Total phenolics, antioxidant properties and quality of fresh-cut onions (*Allium cepa* L.) treated with mild-heat. Food Chem..

[22] Zhiheng L., Chunji L., Ping C., Guohui Y. (2022). Rhodotorula mucilaginosa alternative sources of natural carotenoids, lipids, and enzymes for industrial use. Heliyon.

[23] Wang C., Hsueh P., Chen F., Lee W. (2019). Breakthrough fungemia caused by *Rhodotorula mucilaginosa* during anidulafungin therapy. J. Microbiol. Immunol. Infect..

[24] Lübeck M., Lübeck P.S. (2019). Application of lactic acid bacteria in green biorefineries. FEMS Microbiol..

[25] Singhvi M., Zendo T., Sonomoto K. (2018). Free lactic acid production under acidic conditions by lactic acid bacteriastrains: Challenges and future prospects. Appl. Microbiol. Biotechnol..

[26] Pohanka M. (2020). D-lactic acid as a metabolite: Toxicology, diagnosis, and detection. BioMed Res. Int..

[27] Raj T., Chandrasekhar K., Naresh A., Kim S.H. (2022). Recent biotechnological trends in lactic acid bacterial fermentation for food processing industries. Syst. Microbiol. Biomanuf..

[28] Kellil A., Grigorakis S., Loupassaki S., Makris D.P. (2021). Empirical kinetic modelling and mechanisms of quercetin thermal degradation in aqueous model systems: Effect of pH and addition of antioxidants. Appl. Sci..

[29] Rohn S., Buchner N., Driemel G., Rauser M., Kroh L.W. (2007). Thermal degradation of onion quercetin glucosides under roasting conditions. J. Agric. Food Chem..

[30] Grzelak-Błaszczyk K., Czarnecki A., Klewicki R., Grzegorzewska M., Klewicka E. (2023). Lactic acid fermentation of osmo-dehydrated onion. Food Chem..

